# The Faculty of the Investigator

**Published:** 1908-06-20

**Authors:** 


### The Faculty of the Investigator.

It is not a little overlooked that the mental endowment which enabled Galen to guess correctly at the renal function, Harvey to reconstruct in imagination the path of the circulation, and Addison to group certain symptoms into a constant pathological entity; by virtue of which Helmholtz was imbued with the fine certainty that his rough model of an ophthalmoscope must succeed in its projected function, and Lister with the assurance, "clear as daylight," of the vast bearing of Pasteur's discoveries on surgery, is just the same in kind as the philosophical genius of a Newton, a Kepler, or a Darwin. In both groups we see the faculty of finding "the one in the many" and that power of rapid appreciation of true analogy which, according to Lotze, is responsible for every successful hypothesis. It is not unusual, however, to hear some decry the use of hypothesis altogether, maintaining that one should collect facts without preconceived ideas. From this it would appear as if all that is necessary to discovery is abundant material and the ability to recognise it. A little sifting of results, a little experiment, a little procedure by the method of trial and error will do the rest. These Baconian principles of pure induction answer very well in some simple branches of clinical work. The physician who records carefully during a lifetime of practice the varieties, say, of an exanthematous rash, renders much service to diagnosis; the bold surgeon who first ascertains that removal of some diseased organ does not make life impossible, or that a great deal of the bony framework of a part can be cut away without risk of its collapse, certainly widens the scope of treatment. But for really heuristic contributions to science, medical or otherwise, lengthy experience and highly technical training are required much less than a peculiar cast of mind—the instinct for detecting correlation in natural phenomena, and the resultant power of devising a plan of investigation which shall elucidate matters to the utmost degree possible and at the minimum cost in time and labour. Thus did Faraday, as Jevons has pointed out, make the most extensive additions to human knowledge without passing beyond common arithmetic; and largely thus, too, could Pasteur carry out a successful investigation into silkworm disease, although dependent at first on others for elementary information on his subject. Many details of the history of medical progress show that insight much less than Pasteur's will more than make up for comparative unfamiliarity with a problem. To take an instance, no more than fifty years back, when recognised authorities on consumption were treating their patients within closed windows, a general practitioner was inaugurating the first sanatorium.

What, then, is the secret of this power of correct theorising? The logicians cannot make much of it; it lies, they say, quite outside their province. They may analyse later stages of the process of inductive reasoning, the formation of logical deductions and the careful verifications; but for the first, indispensable, spontaneous, intuitive perception—the creative, conceptive, intellectual act—they can give no rules. But it is allowed that the faculty of generalising originally and correctly is a sign of the highest philosophical power, almost involuntary, though it be. It is the mark of a great mind, for instance, not only to accomplish much itself, but also to inspire others with ideas which may be fruitfully worked out; and this is a respect in which, on the whole, our medical teachers are lacking. For various reasons, in their choice not enough account is taken of possession of this rare reconstructive power, this understanding of (almost sympathy with) the processes of Nature—which is well-nigh as necessary to the designing of the best form of research as sure aiming to effective rifle shooting; since, as Aristotle puts it, the starting point or principle is more than half the whole matter, many of the points of inquiry coming simultaneously into view thereby. What is needed for the advancement of medical learning is not so much expertness in laboratory technique or in refinements of clinical examination as innate ability for the real "scientific use of the imagination." Unfortunately this latter may easily escape current examinational tests, and is not likely, at any rate at first, to bring unmixed joy to its possessor.

